# Sclerosing Angiomatoid Nodular Transformation of the Spleen: Multimodality Imaging Features

**DOI:** 10.7759/cureus.83488

**Published:** 2025-05-05

**Authors:** Burhan Vural, Ihsaniye Suer Dogan, Berna Turhan, Sümeyya Duran Kaymak, Mustafa Hulusi Kurt, Rasime Pelin Kavak

**Affiliations:** 1 Radiology, Ministry of Health Etlik City Hospital, Department of Radiology, Ankara, TUR

**Keywords:** computed tomography (ct) imaging, magnetic resonance imaging(mri), sclerosing angiomatoid nodular transformation, spleen, ultrasound imaging

## Abstract

Sclerosing angiomatoid nodular transformation (SANT) of the spleen is a rare benign lesion. This report presents the case of a middle-aged woman with asymptomatic SANT of the spleen, complicated by metastatic papillary thyroid carcinoma and an undiagnosed splenic mass. The condition was effectively managed through laparoscopic splenectomy, with a definitive diagnosis confirmed postoperatively. This case aims to contribute to enhancing the differential diagnosis of SANT by highlighting its radiological features, particularly in patients undergoing follow-up for malignancy.

## Introduction

Sclerosing angiomatoid nodular transformation (SANT) is a reactive, non-neoplastic, rare vascular lesion of the spleen. It has previously been diagnosed as cord capillary hemangioma and splenic hamartoma [1]. Even though Krishnan first reported it in 1993, the term was officially defined by Martel et al. in 2004 [2].

The pathogenesis of SANT remains poorly understood. It is hypothesized to represent the final phase of a sequence of processes involving inflammatory pseudotumor formation, alteration of the red pulp architecture, and excessive proliferation of non-neoplastic stromal components. Additionally, its development has been linked to Epstein-Barr virus (EBV) infection and IgG4-related sclerosing disease, suggesting a multifactorial etiology [3].

It predominantly affects individuals between the ages of 30 and 60, with a higher incidence reported in females. Given its rarity and the absence of specific clinical manifestations, the preoperative diagnosis of SANT poses significant challenges [4].

In most cases, SANT is identified incidentally during routine follow-up imaging conducted for unrelated pathologies. Such lesions may occasionally be misinterpreted as metastases. When a splenic lesion is detected through imaging modalities like computed tomography (CT), differential diagnoses should include hemangioma, hamartoma, inflammatory pseudotumor, lymphoma, angiosarcoma, littoral angioma, and metastases. The definitive diagnosis of SANT requires a tissue sample for histopathological evaluation and immunohistochemical analysis, ensuring accurate identification of the lesion [5].

Radiologists may often misdiagnose SANT as other splenic tumors due to a limited understanding of its distinct features. Thus, a comprehensive knowledge of the characteristic imaging findings of SANT is crucial to accurately differentiate it from other splenic tumors and reduce diagnostic inaccuracies.

## Case presentation

A 56-year-old female patient presented to our hospital with complaints of swelling on the left side of the neck. She did not report any additional symptoms. Her medical history revealed a total thyroidectomy performed five years ago due to papillary thyroid carcinoma, with no other notable medical history. During physical examination, a mobile mass measuring approximately 15 mm was palpated at level 3 of the left cervical triangle. No additional remarkable findings were observed. In the complete blood count and hemogram results, all parameters were within normal limits except for AST (aspartate aminotransferase) and ALT (alanine aminotransferase, both of which were slightly elevated (Table 1).

**Table 1:** Blood test analysis Slightly elevated aspartate aminotransferase (AST) and alanine aminotransferase (ALT).

Blood test	Patient value	Units	Range
Aspartate aminotransferase (AST)	35	U/L	< 32
Alanine aminotransferase (ALT)	43	U/L	< 33

A superficial ultrasonographic examination of the patient's neck revealed multiple lymphadenopathies, the largest measuring 16x11 mm. The biopsy of the lymphadenopathy confirmed the presence of metastatic papillary thyroid carcinoma, and a left neck lymph node dissection was planned for the patient. As part of the preoperative evaluation, complete abdominal ultrasonography was performed to investigate the mild elevation in liver function tests observed in routine biochemical results.

**Figure 1:**
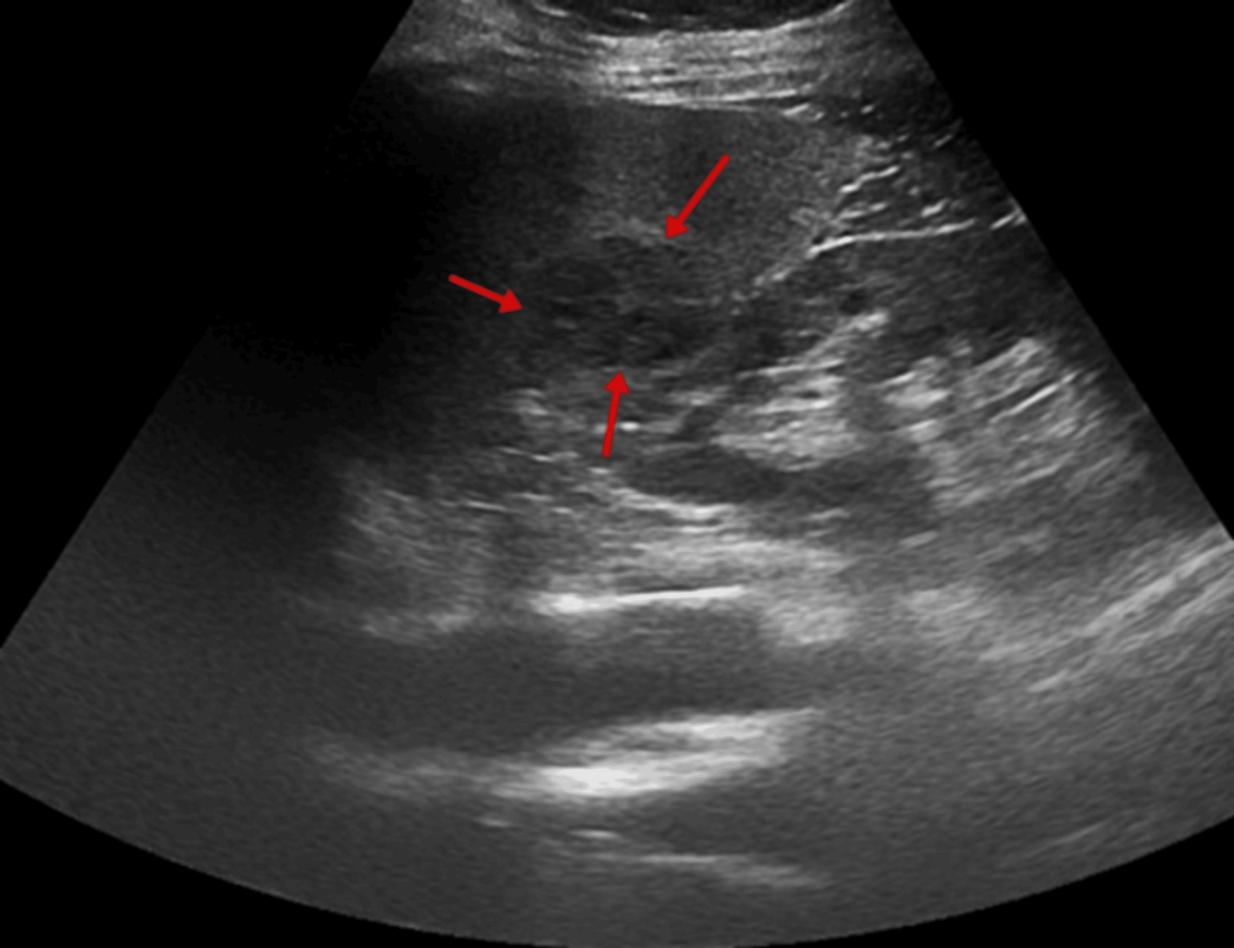
Gray-scale axial abdominal ultrasonography examination At the level of the splenic hilum, a nodular lesion measuring approximately 26x29 mm with mildly lobulated contours is observed. It appears iso-hypoechoic compared to the surrounding splenic parenchyma (red arrows).

Abdominal ultrasonography revealed an increase in liver size, which was consistent with Grade 2 steatosis. Additionally, a heterogeneous hypoechoic mass lesion measuring approximately 29x26 mm with mildly lobulated contours was identified at the level of the splenic hilum (Figure 1). The imaging findings of the lesion were highly indicative of sclerosing angiomatoid nodular transformation (SANT). Given the patient's history of malignancy, laparoscopic splenectomy was performed. Histopathological analysis of the resected mass confirmed the diagnosis of SANT.

In this case, the patient with a history of malignancy underwent contrast-enhanced dynamic computed tomography and magnetic resonance imaging. The purpose of these procedures was twofold. First, they were intended to characterize the incidentally detected splenic lesion. Second, they sought to assess it for possible metastasis.

**Figure 2:**
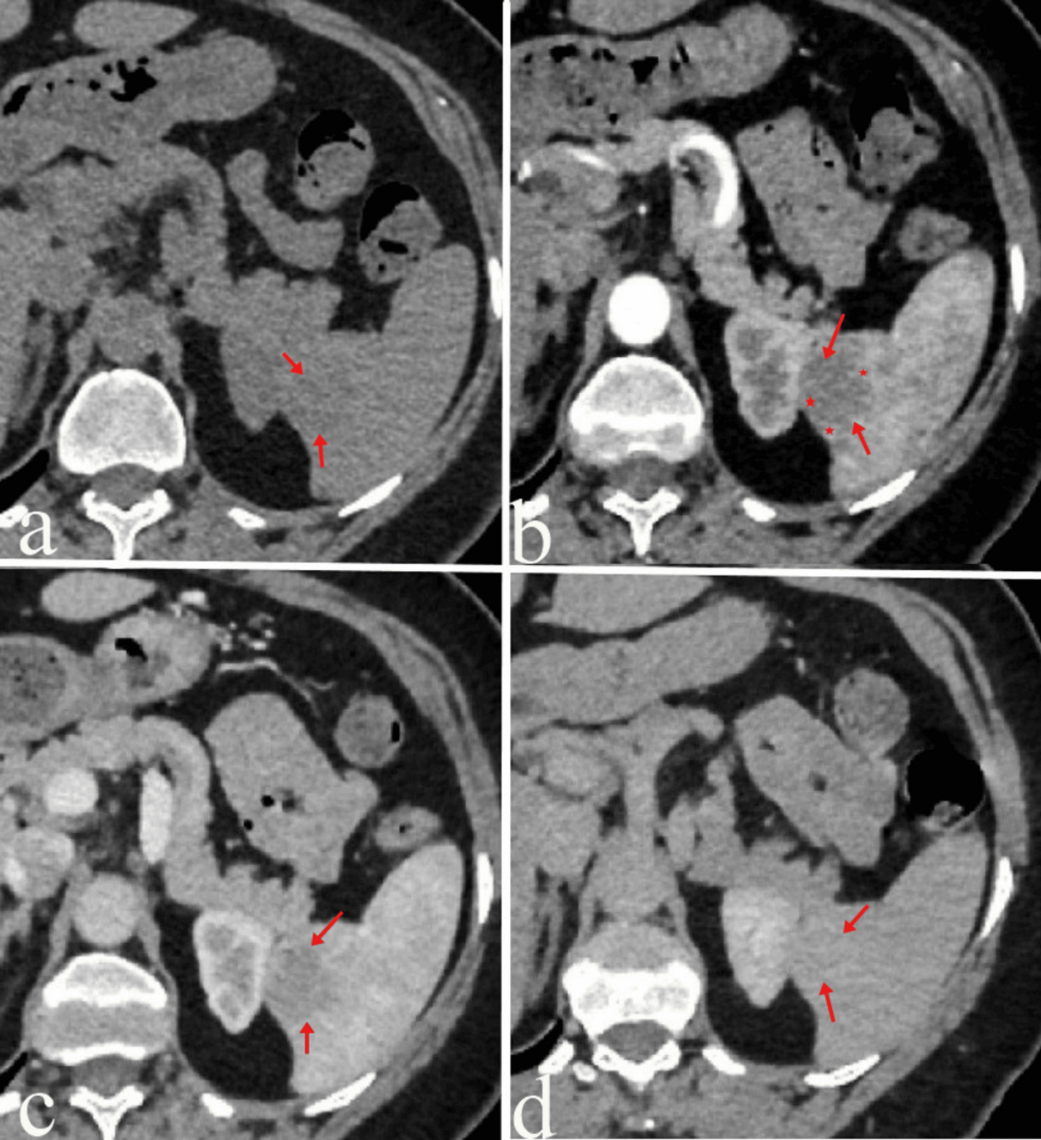
Dynamic contrast-enhanced axial CT examination On non-contrast axial CT imaging (a), a hypodense mass lesion measuring 26x20 mm with mildly lobulated contours is observed in the inferior spleen (red arrows). On post-contrast images (red arrows), the lesion demonstrates peripheral nodular enhancement during the arterial phase (b) (red asterisks) and progressive centripetal enhancement during the portal phase (c). In the late venous phase images (d), the lesion shows enhancement similar to the surrounding splenic parenchyma.

Dynamic computed tomography (CT) revealed a lesion measuring approximately 26x20 mm in the inferior region of the spleen. The lesion appeared iso-hypodense compared to the splenic parenchyma on non-contrast images. The post-contrast series demonstrated progressive contrast enhancement during the arterial and portal phases, but no significant enhancement was observed in the late venous phase (Figure 2).

**Figure 3:**
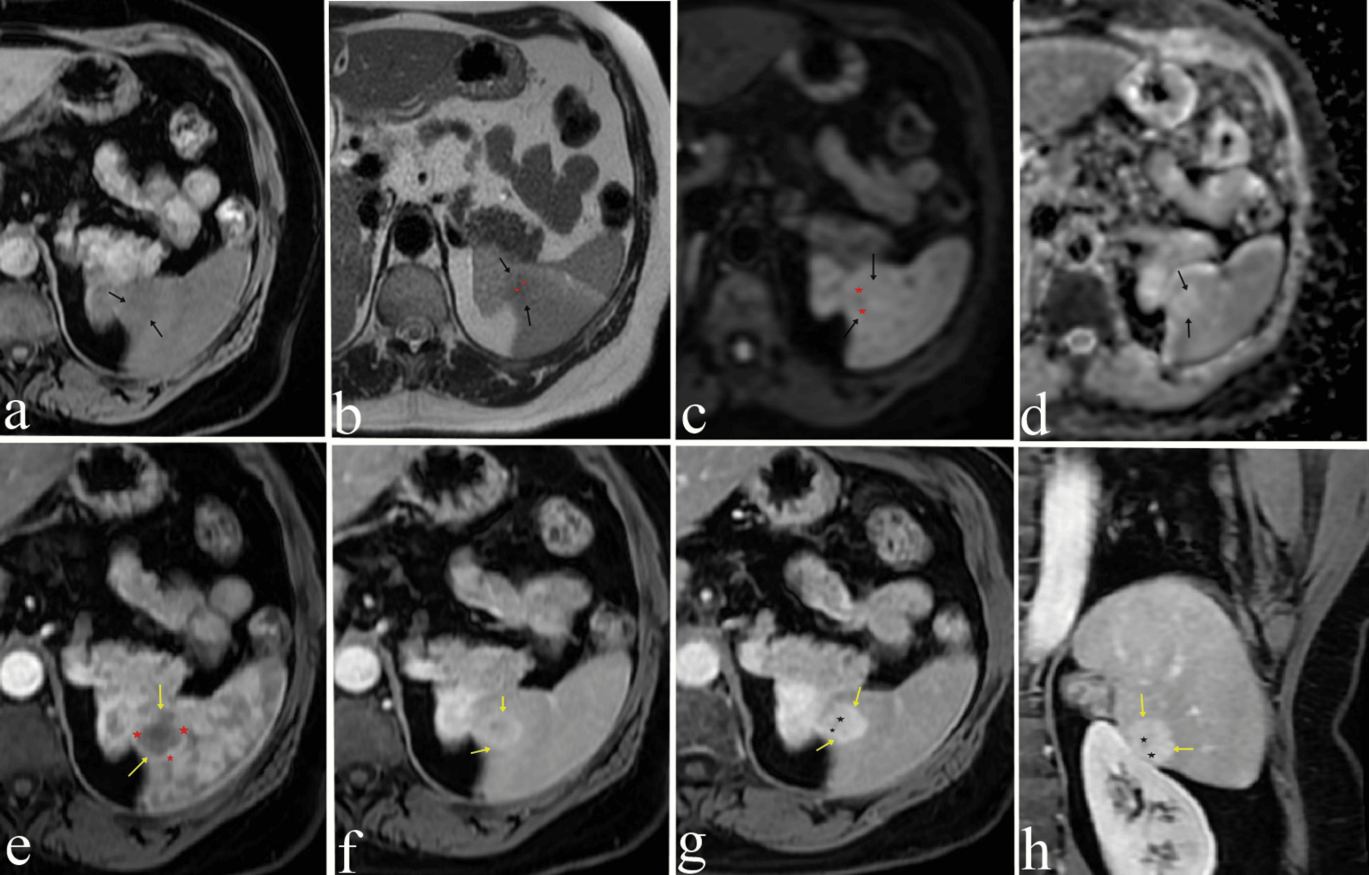
Dynamic MRI examination On non-contrast fat-suppressed T1-weighted axial imaging (a) and T2-weighted axial imaging (b), the lesion (black arrows) demonstrates iso-hypointense signal characteristics. On diffusion-weighted imaging (c) and ADC imaging (d), no significant diffusion restriction is observed. In T2-weighted imaging (b) and diffusion-weighted imaging (c), a centrally located hypointense scar (red asterisks) is noted. Following the administration of contrast material, on arterial T1-weighted imaging (e), the lesion (yellow arrows) demonstrates weak peripheral enhancement (red asterisks) compared to the splenic parenchyma. On post-contrast portal venous phase (f), delayed venous phase axial (g), and coronal (h) images, the lesion exhibits progressive centripetal enhancement (yellow arrows). In delayed venous phase images (g, h), hypointense signal characteristics consistent with a central scar are observed (black arrows).

Dynamic magnetic resonance imaging (MRI) revealed a mass characterized by iso-hypointense signals on T2-weighted images, with central scar tissue observed. The lesion showed no evidence of diffusion restriction (Figure 3).

The imaging findings of the lesion were highly indicative of sclerosing angiomatoid nodular transformation (SANT). Given the patient's history of malignancy, laparoscopic splenectomy was performed. Histopathological analysis of the resected mass confirmed the diagnosis of SANT. Microscopically, the lesion was characterized by multiple angiomatoid nodules composed of slit-like, capillary-sized, and sinusoid-like vascular spaces, surrounded by dense fibrosclerotic stroma.

Four weeks after surgery, the patient showed no postoperative complications, and no signs of recurrence or metastasis were detected. The patient continues to undergo routine follow-up.

## Discussion

Sclerosing angiomatoid nodular transformation (SANT) is a rare, benign vascular lesion of the spleen with an uncertain etiology [6,7]. The pathogenesis of sclerosing angiomatoid nodular transformation (SANT) remains a subject of considerable debate. Proposed theories include abnormal hamartomatous transformation of the splenic red pulp, driven by excessive non-neoplastic stromal proliferation, as well as potential associations with Epstein-Barr virus and IgG4-related disease. Despite these hypotheses, the exact mechanisms underlying SANT remain elusive [8,9]. The majority of patients were female, accounting for 52.1% of the cohort, with a median age of 46 years [10].

SANTs are typically asymptomatic, with lesions often identified incidentally during imaging performed for unrelated conditions or during intra-abdominal surgery. However, in the minority of symptomatic cases, abdominal pain is the most frequently reported symptom, occurring in approximately 25.8% of patients. Additional symptoms reported in the literature include pelvic or flank pain, vomiting, constitutional symptoms such as fever, weight loss, and night sweats, as well as hematologic abnormalities like thrombocytopenia, pancytopenia, and anemia, alongside splenomegaly [11].

Imaging modalities, such as computed tomography (CT) and magnetic resonance imaging (MRI), offer valuable diagnostic insights into sclerosing angiomatoid nodular transformation (SANT). However, the lack of specificity in these techniques hinders their ability to differentiate SANT reliably from other vascular lesions, thereby emphasizing the need for histopathologic examination as the definitive diagnostic approach [12].

The sonographic characteristics of sclerosing angiomatoid nodular transformation (SANT) have been described in only 14 studies, with the most commonly reported characteristic being a solitary, heterogeneously hypoechoic mass [13].

Most studies provide imaging data on computed tomography (CT), where sclerosing angiomatoid nodular transformation (SANT) typically appears as a hypodense, solitary splenic mass in 94% (45/48) of cases. Additionally, 93% (27/29) of cases demonstrate peripheral enhancement with progressive centripetal filling during the delayed phase. Certain studies characterize the enhancement pattern of sclerosing angiomatoid nodular transformation (SANT) as a 'spoke-wheel' configuration. This imaging finding is consistent with a central stellate fibrous stroma, characterized by the presence of fibrous septa that radiate outward, creating a clear delineation between angiomatoid nodules [14].

On MRI, they are heterogeneous and hypo- to isointense on T1-weighted imaging, while they are hypointense on T2-weighted imaging. After the administration of contrast medium, a similar enhancement to that of CT scans is found, with a central hypoenhancing stellate scar. Recent literature reviews indicate that approximately 51.6% of SANT cases exhibit hypointensity on T2-weighted imaging (T2WI), demonstrating better sensitivity than the "spoke-wheel" pattern, which is observed in 48% of cases. On T2WI, most sclerosing angiomatoid nodular transformations (SANTs) display hypointense signal characteristics, with a more pronounced central hypointensity due to their fibrous composition and a higher proportion of fibrous content. Some SANTs demonstrate peripheral hyperintensity with central hypointensity, accompanied by hypointense radiation bands that correspond to a central stellate fibrous stroma and fibrous septa. A central scar may be seen on T1- and T2-WI, with a prevalence of 26% among cases examined [15].

Diffusion-weighted imaging (DWI) is highly sensitive in detecting malignant tumors, as restricted or impeded diffusion is characteristic of malignancies with high cellularity. However, distinguishing benign splenic tumors from malignant ones remains challenging, given that the normal spleen exhibits the highest degree of restricted diffusion among all solid abdominal organs. Yoshimura et al. proposed that multinodular hyperintense areas and fibrotic hypointense regions, which are easily observed on DWI, may serve as distinguishing features of certain splenic lesions [16].

The differential diagnosis of sclerosing angiomatoid nodular transformation (SANT) (Table 2) encompasses various benign and malignant lesions, including hamartomas, hemangiomas, littoral cell angiomas, metastatic tumors, angiosarcomas, and lymphoma. Although radiological differentiation can be challenging, certain characteristics may facilitate a more definitive diagnosis. For instance, hemangiomas and hamartomas typically exhibit hyperintensity on T2-weighted imaging (T2WI), distinguishing them from SANT. The absence of splenomegaly and abdominal lymphadenopathy further aids in differentiating SANT from lymphoma. While innumerable lesions are characteristic of littoral cell angiomas, SANT is generally identified as a solitary lesion. Identifying a primary malignancy is crucial in diagnosing metastatic splenic lesions. Angiosarcoma, due to its aggressive nature, frequently presents with necrotic regions and distant metastases [17].

**Table 2:** Radiological differential diagnosis of solid splenic lesions

Solid Splenic Lesions – Radiologic Differential Diagnosis Features		
Lesion	Nature	Key Radiologic Features
Hemangioma	Benign	T2 hyperintense, similar to liver hemangioma. Doppler US: low flow. CT/MRI: immediate homogeneous or progressive delayed enhancement
Hamartoma	Benign	Solitary, well-circumscribed lesion. US: hypoechoic with increased vascularity. Early arterial enhancement, progressively matches splenic parenchyma
SANT (Sclerosing Angiomatoid Nodular Transformation)	Benign	Solitary, well-defined mass. Characteristic spoke-wheel enhancement pattern. MRI: T2 hypointense fibrous bands, signal loss due to hemosiderin
Angiosarcoma	Malignant	Irregular, heterogeneous, poorly defined nodular masses. Diffuse splenomegaly. Hemorrhage and necrosis are common. Often heterogeneous enhancement
Lymphoma	Malignant	Most common splenic malignancy (usually secondary). Variable appearance: splenomegaly, diffuse or focal lesions. Homogeneous, hypoechoic/hypodense. Restricted diffusion and FDG PET/CT activity
Inflammatory Pseudotumor (IPT)	Benign	Well-circumscribed mass. Hypoechoic, hypodense central fibrous scar: T2 hypointense with progressive enhancement
Granulomatous Disease (e.g., Sarcoidosis)	Benign	Numerous tiny hypoechoic/hypodense nodules MRI: low signal intensity on all sequences, best seen on T2 fat-suppressed and postcontrast images
Littoral Cell Angioma (LCA)	Benign	Multiple nodules with progressive enhancement. Variable T2 signal
Metastases	Malignant	Rare; typically in patients with known disseminated malignancy. Variable appearance depending on the primary tumor origin

In our case, the patient exhibited no additional complaints other than neck swelling. The absence of diffusion restriction in the splenic lesion on diffusion-weighted imaging (DWI), alongside the presence of a central scar on T2-weighted images, lowered the probability of metastasis. In the literature, cases of splenic metastasis due to papillary thyroid carcinoma have been reported, though these cases are exceedingly rare [18]. However, as metastasis could not be conclusively excluded in this patient with metastatic papillary carcinoma, a laparoscopic splenectomy was performed. Pathological examination of the specimen revealed findings consistent with sclerosing angiomatoid nodular transformation (SANT).

Splenectomy can be both diagnostic and curative [19]. The integration of multiple imaging modalities significantly enhances the likelihood of achieving a precise, non-invasive diagnosis, thereby obviating the need for biopsies or splenectomy. A meta-analysis by Aziret et al. showed that a definitive preoperative diagnosis of sclerosing angiomatoid nodular transformation (SANT) could be made in 62 of 230 patients [20].

In the context of differential diagnoses, particularly for splenic lesions where metastasis cannot be ruled out, sclerosing angiomatoid nodular transformation (SANT) should be regarded as a critical consideration.

## Conclusions

The imaging characteristics of sclerosing angiomatoid nodular transformation (SANT) on ultrasound and computed tomography (CT) are relatively nonspecific. However, the diagnosis of SANT can be more confidently proposed when magnetic resonance imaging (MRI) identifies a lobulated lesion with smooth margins. This lesion exhibits isointensity on T1-weighted imaging and markedly low signal intensity on T2-weighted imaging compared to normal splenic tissue. Characteristic features such as peripheral arterial enhancement with delayed centripetal filling or a spoke-wheel enhancement pattern, combined with moderate or heterogeneous uptake on FDG-PET imaging, can substantially reinforce the diagnostic likelihood of SANT. The absence of malignant indicators like washout, infiltrative growth, and adenopathy further strengthens the possibility of a SANT diagnosis.
